# 3D Latent Diffusion Model for MR-Only Radiotherapy: Accurate and Consistent Synthetic CT Generation

**DOI:** 10.3390/diagnostics15233010

**Published:** 2025-11-26

**Authors:** Mohammed A. Mahdi, Mohammed Al-Shalabi, Ehab T. Alnfrawy, Reda Elbarougy, Muhammad Usman Hadi, Rao Faizan Ali

**Affiliations:** 1 Information and Computer Science Department, College of Computer Science and Engineering, University of Ha’il, Ha’il 55476, Saudi Arabia; m.mahdi@uoh.edu.sa (M.A.M.); moh.alshalbi@uoh.edu.sa (M.A.-S.); 2 Information Security Department, College of Computer Science and Engineering, University of Ha’il, Ha’il 55476, Saudi Arabia; alnfrawy@gmail.com; 3 Artificial Intelligence and Data Science Department, College of Computer Science and Engineering, University of Ha’il, Ha’il 55476, Saudi Arabia; reda.elsayed@uoh.edu.sa; 4 School of Engineering, Ulster University, Belfast BT15 1AP, UK; m.hadi@ulster.ac.uk; 5 School of Computing, University of Kent, Canterbury CT2 7NZ, UK

**Keywords:** latent diffusion models, medical image synthesis, MRI-to-CT translation, 3D volumetric imaging, generative models, synthetic CT, radiotherapy planning

## Abstract

**Background**: The clinical imperative to reduce patient ionizing radiation exposure during diagnosis and treatment planning necessitates robust, high-fidelity synthetic imaging solutions. Current cross-modal synthesis techniques, primarily based on GANs and deterministic CNNs, exhibit instability and critical errors in modeling high-contrast tissues, thereby hindering their reliability for safety-critical applications such as radiotherapy. Objectives: Our primary objective was to develop a stable, high accuracy framework for 3D Magnetic Resonance Imaging (MRI) to Computed Tomography (CT) synthesis capable of generating clinically equivalent synthetic CTs (sCTs) across multiple anatomical sites. **Methods:** We introduce a novel 3D Latent Diffusion Model (3DLDM) that operates in a compressed latent space, mitigating the computational burden of 3D diffusion while leveraging the stability of the denoising objective. **Results:** Across the Head & Neck, Thorax, and Abdomen, the 3DLDM achieved a Mean Absolute Error (MAE) of 56.44 Hounsfield Units (HU). This result demonstrates a significant 3.63% reduction in overall error compared to the strongest adversarial baseline, CycleGAN (MAE = 60.07 HU, *p* < 0.05), a 10.76% reduction compared to NNUNet (MAE = 67.20 HU, *p* < 0.01), and a 20.79% reduction compared to the transformer-based SwinUNeTr (MAE = 77.23 HU, *p* < 0.0001). The model also achieved the highest structural similarity (SSIM = 0.885 ± 0.031), significantly exceeding SwinUNeTr (*p* < 0.0001), NNUNet (*p* < 0.01), and Pix2Pix (*p* < 0.0001). Likewise, the 3D-LDM achieved the highest peak signal-to-noise ratio (PSNR = 29.73 ± 1.60 dB), with statistically significant gains over CycleGAN (*p* < 0.01), NNUNet (*p* < 0.001), and SwinUNeTr (*p* < 0.0001). **Conclusions:** This work validates a scalable, accurate approach for volumetric synthesis, positioning the 3DLDM to enable MR-only radiotherapy planning and accelerate radiation-free multi-modal imaging in the clinic.

## 1. Introduction

Medical imaging, such as Magnetic Resonance Imaging (MRI) and Computed Tomography (CT) is foundational to modern clinical practice [1], enabling non-invasive diagnosis [2], treatment planning [3], and disease monitoring [4,5]. However, the acquisition of high-quality, diverse medical datasets is often hampered by significant challenges, including patient privacy concerns, data scarcity, and the high cost of acquisition [6,7]. These constraints create bottlenecks for developing robust machine learning systems—particularly in rare disease settings, radiotherapy applications, and scenario-specific data augmentation tasks [8,9]. Consequently, medical image synthesis has emerged as a powerful strategy for generating realistic substitute datasets that reduce reliance on protected health data while supporting supervised learning, anomaly detection, and physician training [10,11,12].

Prior research in MRI-to-CT translation has explored a spectrum of techniques, ranging from classical atlas-based methods to modern deep learning (DL) approaches. Early methods relied on registering an MRI to a pre-existing CT atlas, but this proved inadequate for individual patient variations and pathological conditions [13,14]. The advent of deep convolutional neural networks (CNNs) and Generative Adversarial Networks (GANs) marked a significant leap, providing an end-to-end framework for image synthesis [15,16,17]. While GAN-based models have demonstrated impressive two-dimensional results, they often suffer from training instability and are prone to generating clinically implausible artifacts and “hallucinations” [18,19]. Moreover, adapting these methods to the high-dimensional complexity of 3D volumetric data remains a substantial challenge, often leading to a compromise between computational efficiency and the preservation of anatomical consistency and fine-grained detail across slices [20].

Despite these advancements, a critical research gap persists: a robust, scalable, and clinically reliable method for 3D MRI-to-CT synthesis that can accurately model complex anatomical structures while maintaining high clinical fidelity and generalizability across diverse patient populations [13,14,19]. Current techniques struggle to capture the full spectrum of anatomical variability and often fail to produce synthesized volumes that are metrically and structurally equivalent to real CT scans [20]. A central challenge lies in designing a generative framework capable of learning the intricate cross-modal mapping in high-dimensional space without sacrificing computational tractability or introducing artifacts that could limit diagnostic confidence [21,22].

To address this, we introduce an approach for high-fidelity 3D MRI-to-CT synthesis using a Latent Diffusion Model (LDM). Our method represents a paradigm shift from conventional generative models by leveraging the superior denoising and generative capabilities of diffusion models (DMs). The key innovation lies in our two-stage architecture: an autoencoder first compresses the high-resolution 3D MRI volumes into a compact, semantically rich latent space, where a diffusion model is then trained to learn the modality translation. This latent space design enables us to efficiently process entire 3D volumes while the iterative denoising process ensures the generation of diverse, high-quality CT outputs with remarkable realism and structural integrity. Our contributions are multi-faceted: we propose 3D LDM for clinical MRI-to-CT synthesis with a region-agnostic latent training strategy, validated with rigorous qualitative and quantitative metrics; we demonstrate unprecedented anatomical accuracy and consistency, surpassing state-of-the-art (GANs, SwinUNeTr, NNUNet); and we show exceptional generalization across three different patient cohorts.

The remainder of this paper is structured as follows: Section 2 provides an overview of related work in medical image synthesis. Section 3 details our proposed LDM architecture and the two-stage training methodology, including the dataset, implementation details, and evaluation metrics. Section 4 presents a comprehensive analysis of our qualitative and quantitative results, including a comparison with baseline models. Finally, Section 5 provides a concluding summary of our work and discusses potential avenues for future research.

## 2. Related Work

The synthesis of sCT from MRI has evolved from classical atlas-based registration techniques to modern deep generative models [23]. Early CNN-based architectures, including U-Net variants [24,25], established the feasibility of supervised MRI-to-CT synthesis but often produced blurred soft-tissue interfaces and struggled with bone-air boundaries critical for dose calculation.

To overcome these limitations, GANs were adopted, introducing an adversarial loss to encourage the generator to produce more realistic outputs that are difficult for a discriminator to distinguish from real CTs. Liu et al. [26] proposed a Multi-Cycle GAN for head-and-neck MRI-to-CT synthesis, which incorporated a pseudo-cycle consistency module to enhance generation stability, a domain control module to improve structural fidelity, and a novel Z-Net generator to better preserve anatomical details. While GANs demonstrated improved sharpness and detail compared to earlier CNN-based methods, they remain prone to training instability and mode collapse, which can result in limited output diversity and the generation of clinically implausible artifacts.

For unpaired data, which is more readily available, CycleGAN became a popular solution. Zhu et al. [27] applied a CycleGAN with an added structure-consistency loss to synthesize brain CTs from unpaired MRIs. Their method demonstrated the ability to learn cross-modal mapping without one-to-one pixel correspondence, a significant practical advantage. However, unpaired methods often struggle with hallucinating anatomical features, as the cycle-consistency constraint does not guarantee fine-grained structural fidelity. Furthermore, GANs often fail to capture the full data distribution, leading to limited output diversity and poor generalization to unseen anatomical variations or pathology [28].

More recently, transformers have been integrated into medical image synthesis pipelines to better model long-range dependencies, a known limitation of CNNs [29]. Pan et al. [30] presented a transformer-based improved denoising diffusion probabilistic model (MC-IDDPM) for MRI-to-CT synthesis in brain and prostate datasets. Their approach combined a diffusion framework with a shifted-window transformer network (Swin-VNet) to capture both local detail and global anatomical context. While this work highlights the potential of transformer-based diffusion models for accurate and reliable volumetric synthesis, the high computational burden of 3D diffusion-transformer architectures, even with windowing strategies, remains a barrier to widespread clinical deployment. DMs and, more recently, LDMs [31,32], provide a compelling alternative by combining the superior generative stability of diffusion with latent space compression to reduce computational demands [33]. This LDM paradigm has been successfully applied and clinically validated for sCT generation, achieving high dosimetric accuracy suitable for MR-only radiotherapy planning [34]. The advantages of the LDM include high structural consistency, avoidance of mode collapse, and computational tractability in 3D directly motivate our proposed framework.

## 3. Materials and Methods

**Figure 1:**
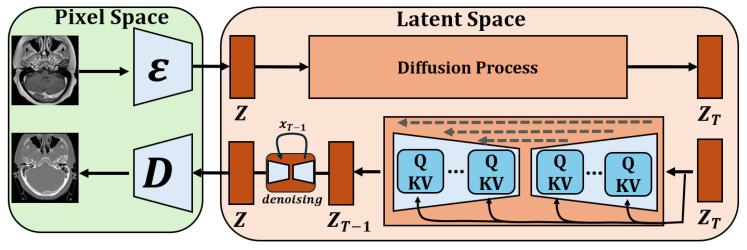
Overview of the 3D Latent Diffusion Model Architecture.

The synthesis of sCT from MRI is achieved through a two-stage 3D LDM, as illustrated in Figure 1. This generative framework is designed to synthesize high-fidelity sCT volumes from their corresponding MRI counterparts by leveraging a compressed latent space to ensure computational efficiency. The entire pipeline, from data preparation to model evaluation, is meticulously detailed in the following sections.

### 3.1. Dataset and Preprocessing

The model was trained, validated, and tested using the SynthRAD2023 Challenge [34], Task 1 dataset, which consists of paired T1-weighted brain MRI and co-registered CT scans and. T1-weighted sequences were used because they are clinically preferred for synthetic CT generation in radiotherapy dose planning. To ensure data consistency and reduce the computational burden, each 3D volume underwent a series of preprocessing steps. First, the anatomical orientation of all images was standardized to the radiological standard (RAS). Subsequently, intensity values were normalized by clipping the images to the range defined by the 0th and 99.5th percentiles and then rescaling them to a [0, 1] range. The images were also resampled to a uniform isotropic voxel spacing of (2.4, 2.4, 2.2) mm using bilinear interpolation. Finally, all volumes were center-cropped to a uniform size of (96, 96, 64) voxels to focus on the anatomical region of interest. The dataset was split into training and testing subsets as summarized in Table 1.

Because CT values span a wide physical range (approximately −1024 to +3000 HU), we applied a region-specific clipping strategy during training to emphasize clinically relevant density ranges and suppress extreme outliers—an approach commonly adopted in radiotherapy dose planning. Specifically, MRI-to-CT pairs were clipped to −400 to 300 HU for the thorax, −180 to 250 HU for the brain, and −180 to 400 HU for the abdomen, reflecting standard clinical windowing presets used for dose calculation. All MRI inputs and CT targets were normalized to [0, 1], and the model was trained to predict normalized CT intensities in this bounded space. During inference, synthetic CT predictions were converted back into Hounsfield Units using an inverse linear transformation, $sCT_{HU}=\hat{x}\left(HU_{\max}^{region}-HU_{\min}^{region}\right)+HU_{\min}^{region}$, where the minimum and maximum correspond to the region-specific clipping ranges used during training.

### 3.2. Model Architecture

The generative pipeline comprises two primary components: an AutoencoderKL and a DiffusionModelUNet. The AutoencoderKL serves as a latent space compression module, converting the high-dimensional volumetric data into a lower-dimensional representation. This VAE-style network features 3D architecture with 1 input channel, 1 output channel, and channels configured at $\left(32,64,64\right)$. Its latent space is defined by 3 channels, enabling it to learn a compact representation of the input volumes. As illustrated in Figure 1, the autoencoder’s encoder $\left(E\right)$ maps the MRI from the pixel space to the latent space, while its decoder $\left(D\right)$ reconstructs the sCT from this latent representation. Crucially, the encoder provides the mean $\left(\mu \right)$ and log variance $\left(\log\sigma^{2}\right)$ of a Gaussian distribution, from which the latent vector z is sampled. The compression into this compact latent space is a key step that dramatically reduces the computational complexity of the subsequent diffusion process, making it feasible for 3D volumetric data.

The core generative engine is a DiffusionModelUNet, which operates exclusively within the compressed latent space. This UNet is designed to reverse the gradual noising process of a diffusion model. Its 3D architecture accepts 3 input and 3 output channels, matching the dimensions of the latent space. With channels at $\left(32,64,64\right)$ and attention at levels (False, True, True), this network is conditioned on the diffusion timestep to predict the noise component. The forward diffusion process adds noise to the latent vector over 1000 timesteps, governed by a scaled linear beta schedule (beta_star t=0.0015, beta_end =0.0195). During inference, this process is reversed, enabling the model to generate a new latent vector from pure noise, which is then decoded to form the final sCT.

### 3.3. Loss Functions and Optimization

The model’s training is a two-stage process. The first stage focuses on training the autoencoder using a composite loss function to ensure accurate and high-fidelity image reconstruction. The total loss for the autoencoder ($L_{\mathrm{autoencoder}}$) is a weighted sum of four components: a reconstruction loss, a perceptual loss, a KL divergence loss, and an adversarial loss. The reconstruction loss ($L_{\mathrm{recon}}$) is calculated using the L1-norm to measure pixel-wise differences:

(1) $$L_{\mathrm{recon}}=∥x-D\left(E\left(x\right)\right){∥}_{1},$$

where x is the original image and $D\left(E\left(x\right)\right)$ is its reconstruction. The perceptual loss ($L_{p}$) uses a pre-trained SqueezeNet to align high-level feature representations:

(2) $$L_{p}=∥\psi \left(x\right)-\psi \left(D\left(E\left(x\right)\right)\right){∥}_{1}$$

where ψ denotes the feature extractor. The KL divergence loss ($L_{kl}$) regularizes the latent space distribution to a standard normal:

(3) $$L_{kl}=\frac{1}{2}\underset{i}{\sum }\left(\mu_{i}^{2}+\sigma_{i}^{2}-\log(-1\right)$$

Lastly, an adversarial loss ($L_{\mathrm{adv}}$), based on the least-squares objective, is introduced to encourage realism, with a warm-up period of 5 epochs. The total loss is defined as:

(4) $$L_{\mathrm{autoencoder}}=L_{\mathrm{recon}}+w_{kl}L_{kl}+w_{p}L_{p}+w_{\mathrm{adv}}L_{\mathrm{adv}}$$

with weights $w_{kl}=1\times 10^{-6},w_{p}=0.001$, and $w_{adv}=0.01$. The second stage trains the diffusion UNet using a simple Mean Squared Error (MSE) loss between the predicted noise ($\epsilon_{\theta }$) and the ground truth noise $\left(\epsilon \right)$, as shown below:

(5) $$L_{\mathrm{diff}}=∥\epsilon -\epsilon_{\theta }{\left(z_{t},t\right)∥}_{2}^{2}$$

where $z_{t}$ is the noisy latent vector at timestep t. Both training stages utilized the Adam optimizer with a learning rate of $1\times 10^{-4}$ and leveraged automatic mixed precision (AMP) for improved training efficiency.

### 3.4. Model Evaluation

To rigorously assess the performance of our 3D LDM, we conducted a comprehensive quantitative evaluation of the sCT volumes. The accuracy of the generated sCTs was benchmarked against the ground-truth CTs using a suite of image similarity metrics, all computed within the dilated body contour masks (B) provided by the SynthRAD2023 Challenge. This approach ensures that our evaluation is focused on clinically relevant anatomical regions, excluding background noise. We used three primary metrics to quantify the fidelity of our generated images: Mean Absolute Error (MAE), Peak Signal-to-Noise Ratio (PSNR), and Structural Similarity Index Measure (SSIM). The Masked MAE was calculated to provide a direct measure of the average absolute voxel-wise difference between the sCT and the reference CT, normalized by the number of voxels within the masked region. This metric is expressed as:

(6) $$\mathrm{MAE}(\mathrm{CT},\mathrm{sCT})=\frac{1}{\left|B\right|}\underset{i\in B}{\sum }\left|{\mathrm{CT}}_{i}-{\mathrm{sCT}}_{i}\right|$$

A lower MAE value indicates a closer correspondence between the synthetic and real CT volumes.

To quantify the ratio of maximum signal intensity to noise, we computed the Masked PSNR. This metric is particularly useful for assessing the generative model’s ability to maintain a high level of image quality relative to the inherent noise. The formula is defined as:

(7) $$\mathrm{PSNR}\left(\mathrm{CT},\mathrm{sCT}\right)=10\log_{10}\left(\frac{Q^{2}}{\frac{1}{\left|B\right|}\sum_{i\in B}{\left({\mathrm{CT}}_{i}-{\mathrm{sCT}}_{i}\right)}^{2}}\right)$$

Here, Q represents the dynamic range of voxel intensities, which was set to $\left[-1024,3000\right]\mathrm{HU}$ for our evaluation. The CT and sCT volumes were clipped to this range before the calculation. A higher PSNR value signifies superior image quality and a lower noise level in the synthesized images.

Finally, we used Masked SSIM to evaluate the preservation of structural integrity. This metric goes beyond simple intensity differences by assessing structural, luminance, and contrast similarities between the sCT and ground-truth CT. The SSIM for a local window centered on voxel i is given by:

(8) $${\mathrm{SSIM}}_{i}\left(\mathrm{CT},\mathrm{sCT}\right)=\frac{\left(2\mu_{{\mathrm{CT}}_{i}}\mu_{{\mathrm{sCT}}_{i}}+c_{1}\right)\left(2\sigma_{\mathrm{CT},{\mathrm{sCT}}_{i}}+c_{2}\right)}{\left(\mu_{{\mathrm{CT}}_{i}}^{2}+\mu_{{\mathrm{sCT}}_{i}}^{2}+c_{1}\right)\left(\sigma_{{\mathrm{CT}}_{i}}^{2}+\sigma_{{\mathrm{sCT}}_{i}}^{2}+c_{2}\right)}$$

where μ and σ are the local mean and standard deviation, respectively, and $\sigma_{\mathrm{CT},\mathrm{sCT}}$ is the covariance within a 7×7×7 window. The constants are defined as $c_{1}={\left(0.01\cdot L\right)}^{2}$ and $c_{2}={\left(0.03\cdot L\right)}^{2}$, where L is the dynamic range of the volumes, adjusted to be non-negative. The final masked SSIM value is the average of the local SSIM scores within the body contour mask:

(9) $$\mathrm{SSIM}(\mathrm{CT},\mathrm{sCT})=\frac{1}{\left|B\right|}\sum_{i\in B}{\mathrm{SSIM}}_{i}\left(\mathrm{CT},\mathrm{sCT}\right)$$

This comprehensive set of metrics provides a robust and multifaceted assessment of our model’s performance, ensuring that both pixel-level accuracy and structural fidelity are captured.

### 3.5. Implementation Details

All models were implemented in PyTorch (v1.13.1 + CUDA 11.7) using MONAI (v1.2.dev2304) and trained on a single NVIDIA A100 GPU (40 GB VRAM). We optimized using Adam (β_1_ = 0.9, β_2_ = 0.999) with an initial learning rate of 1 × 10^−4^ and a cosine annealing schedule. The maximum training length was set to 1000 epochs with a batch size of 2 and a patch size of 96 × 96 × 64 voxels. Early stopping was employed based on validation loss. In practice, most models converged and terminated around ~600 epochs, while nnU-Net converged earlier (~300 epochs), reflecting its strong inductive bias and stabilization behavior. The 96 × 96 × 64 patch size was chosen to balance anatomical context with GPU memory constraints during 3D diffusion training. This resolution is consistent with prior work and allows stable learning while preserving all clinically relevant voxel-level HU transitions. Importantly, patching is used only during training—final synthetic CT volumes are reconstructed at the original full resolution without upsampling from 96 to 256. Therefore, the clinical resolution of the output is not degraded. Data augmentation was applied online using MONAI transforms, including random spatial flips, affine transformations, and intensity perturbations. All experiments were conducted in Python 3.10, with the following key dependencies: PyTorch Ignite 0.4.10, ITK 5.3.0, Nibabel 4.0.2, scikit-image 0.19.3, Pillow 9.3.0, TorchVision 0.14.1, and einops 0.6.0. 

### 3.6. Statistical Analysis

All results are reported with 95% confidence intervals (CI). To evaluate whether the performance differences between the proposed 3D-LDM and the baseline models were statistically significant, paired two-sided *t*-tests were conducted for each metric and anatomical region. Significance levels were defined as follows: *** *p* < 0.0001, ** *p* < 0.01, and * *p* < 0.05, while the absence of a marker indicates no statistically significant difference. These thresholds were applied consistently across all anatomical regions and evaluation metrics.

## 4. Results

This section presents a comprehensive evaluation of our proposed 3DLDM for synthetic CT generation based on MR. Performance is assessed through both quantitative metrics, which compare voxel-wise accuracy and structural similarity against four state-of-the-art baselines, and a qualitative analysis that utilizes error mapping to visually confirm fidelity across diverse anatomical sites. Our findings demonstrate that the 3DLDM consistently achieves superior performance in terms of accuracy, structural preservation, and synthesis robustness, effectively mitigating the common failure modes observed in existing generative architectures.

### 4.1. Quantitative Analysis

The quantitative performance of our proposed 3D 3DLDM was rigorously evaluated against a suite of established baselines: SwinUNeTr, NNUNet, CycleGAN, and Pix2Pix. The analysis, presented through a series of box plots, focuses on three core metrics (MAE, PSNR, and SSIM) across three distinct anatomical regions: Head & Neck (HN), Thorax (TH), and Abdomen (AB). The box plots summarize the distribution of these metrics, with the central black x marking the mean, the central line indicating the average, and the box representing the interquartile range (IQR).

**Figure 2:**
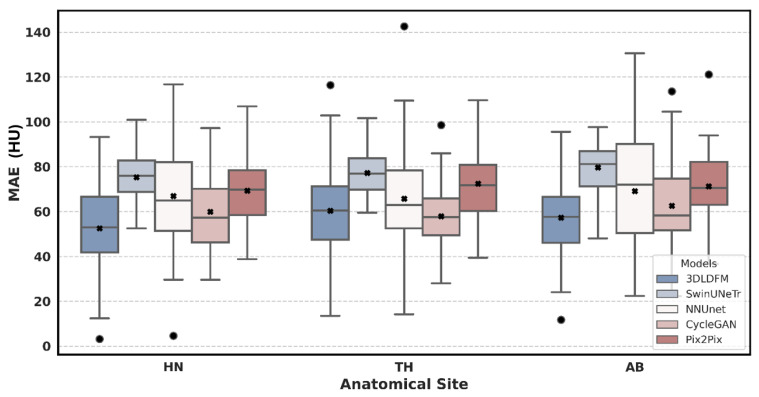
Mean Absolute Error Distribution by Model and Anatomical Site. The circle represents outliers, and the × denotes the mean value.

The distribution of MAE values, a direct measure of voxel-wise intensity accuracy, reveals the superior performance of our 3DLDM across all evaluated anatomical sites. As shown in Figure 2, our model consistently exhibits a lower mean MAE compared to all baselines. Notably, the MAE for the 3DLDM is significantly lower than that of the other models, indicating that our approach produces more accurate synthetic CTs for the majority of cases. These reductions were statistically significant across most comparisons, including SwinUNeTr (*p* < 0.0001), nnU-Net (*p* < 0.01), and Pix2Pix (*p* < 0.0001), and were not significantly different from CycleGAN in some regions (*p* > 0.05). The interquartile range (IQR) for 3DLDM is also markedly narrower and concentrated near the lower error range, indicating greater reliability and reduced variability across patients. For context, recent sCT studies typically report MAE values in the range of 40–80 HU depending on anatomy and acquisition protocol; therefore, the MAE values observed in this work fall within the expected and clinically relevant range. In contrast, models such as nnU-Net and SwinUNeTr show a wider spread of MAE values, reflecting greater variability in synthesis quality. These findings demonstrate the 3DLDM’s ability to learn the complex, non-linear mapping between MRI and CT intensity values and to deliver superior quantitative accuracy across anatomical regions.

**Figure 3:**
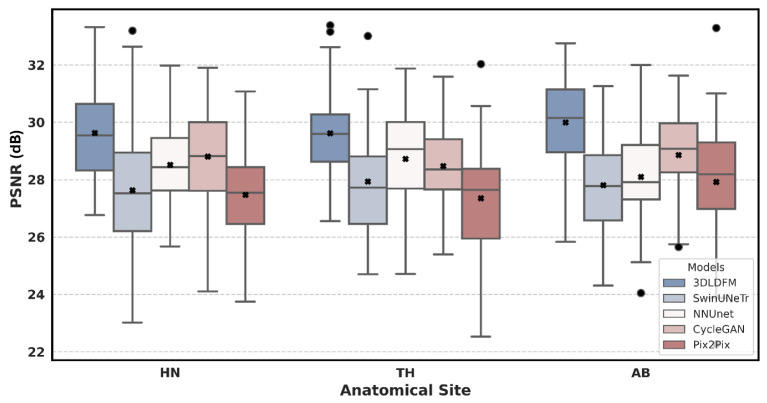
Peak Signal-to-Noise Ratio (PSNR) Distribution by Model and Anatomical Site. The circle represents outliers, and the × denotes the mean value.

An analysis of the PSNR, a key indicator of image quality and clarity, reinforces the findings from the MAE evaluation. As depicted in Figure 3, our 3DLDM consistently achieves the highest mean PSNR across all three anatomical regions. For example, in the Abdomen region, 3D-LDM reached a mean PSNR of 29.99 dB, which was significantly higher than SwinUNeTr (*p* < 0.0001), nnU-Net (*p* < 0.0001), and Pix2Pix (*p* < 0.0001), while outperforming CycleGAN without a statistically significant difference (*p* > 0.05). The box plots for the 3DLDM are shifted upwards, with their central quartiles well above those of the other models. This indicates that our model generates images with a higher signal-to-noise ratio, reflecting sharper details and a reduction in artifacts. While some baselines, such as NNUNet and CycleGAN, demonstrate competitive performance in certain regions, the 3DLDM’s PSNR distribution is consistently superior. This superior image quality can be attributed to the inherent stability of the diffusion process, which effectively suppresses noise and prevents the generation of the spurious details often seen in GAN-based models.

**Figure 4:**
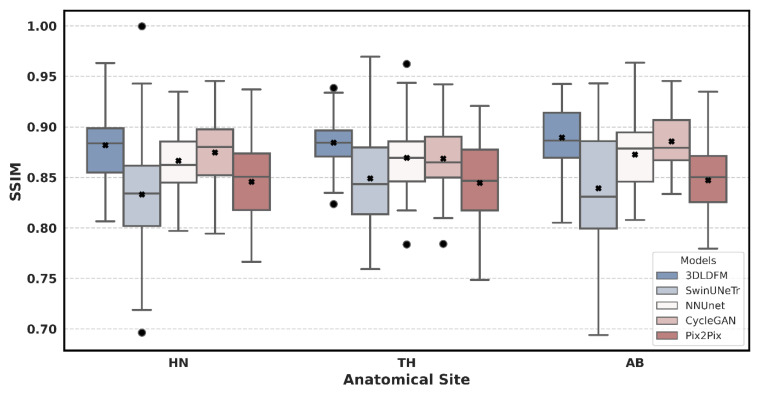
Structural Similarity Index Measure (SSIM) Distribution by Model and Anatomical Site. The circle represents outliers, and the × denotes the mean value.

Beyond simple intensity accuracy, the SSIM metric evaluates the preservation of structural integrity. As shown in Figure 4, our 3DLDM consistently achieves the highest or near-highest SSIM values across all anatomical regions, with mean scores approaching 0.89–0.90, which is close to the theoretical maximum of 1.0. These improvements are statistically significant when compared to SwinUNeTr (*p* < 0.0001), nnU-Net (*p* < 0.01), and Pix2Pix (*p* < 0.0001), and not significantly different from CycleGAN in a small number of cases (*p* > 0.05). The SSIM distributions for 3DLDM are also tightly clustered, indicating robustness and reduced variability across patients. This result is clinically meaningful, as high SSIM values reflect the model’s ability to preserve fine anatomical structures and spatial relationships between voxels, an essential requirement for radiotherapy planning and multi-modal image analysis. In contrast, GAN-based methods often exhibit unpredictable outputs and may hallucinate or distort structural boundaries, whereas the diffusion-based architecture used in 3DLDM provides greater stability, noise suppression, and anatomical reliability. When considered alongside its leading performance in MAE and PSNR, these SSIM findings provide strong evidence that the proposed 3DLDM generates synthetic CT volumes that are not only quantitatively accurate but also structurally faithful and clinically viable.

### 4.2. Assessment and Error Mapping

The visual inspection and quantitative error mapping of the sCT volumes provide compelling qualitative evidence that consistently reinforces our superior quantitative results (MAE, PSNR, SSIM). To ensure a representative analysis across anatomical diversity, three patient cases were selected randomly for visual display from the test set for each anatomical site (Head & Neck, Thorax, and Abdomen). Our 3DLDM demonstrates robust performance across all selected cases, successfully mitigating the critical failure modes, primarily density misestimation, observed in leading baselines.

**Figure 5:**
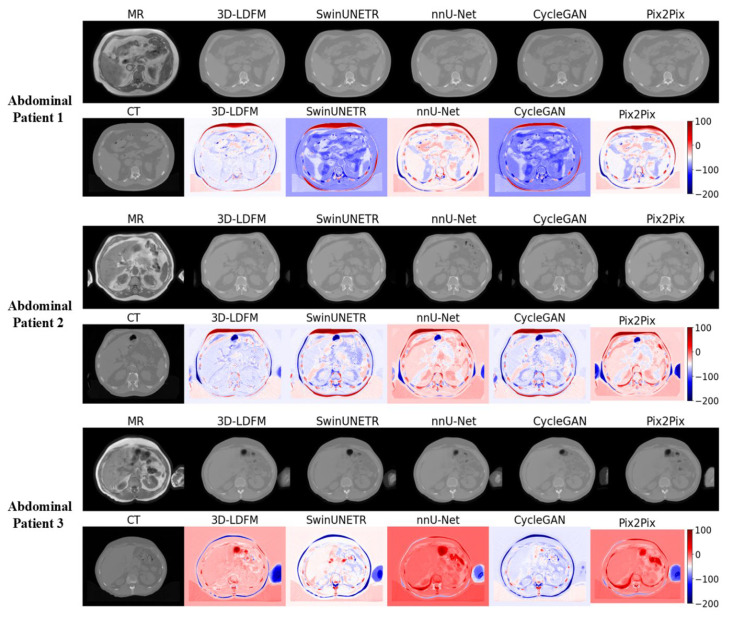
Abdomen (Pelvis/Lower Abdomen Region). Qualitative comparison and error maps for sCT generation in the pelvis/lower abdomen region. Rows show MR input, ground-truth CT, and sCT predictions from multiple methods, followed by HU-difference error maps. 3D-LDFM demonstrates the most accurate soft-tissue modelling, reflected in lower-magnitude and less saturated error maps.

The synthesis results for the Abdomen, shown in Figure 5, confirm the 3DLDM’s high generalizability across various soft-tissue dominated structures and organs. In this region, the primary challenge is distinguishing subtle density variations among different soft tissues (e.g., liver, spleen, fat). Our model consistently produces sCTs that match the soft-tissue contrast of the ground-truth images with high fidelity. The error maps for the 3DLDM are conspicuously cleaner and lighter-toned compared to the baselines. While CycleGAN and Pix2Pix also perform reasonably well in this region compared to the high-contrast bone areas, they still exhibit wider areas of concentrated error, often manifesting as localized patches of misestimated density near organ boundaries. The NNUNet, while demonstrating a strong backbone, still suffers from diffuse error across the soft tissue, suggesting a slight blurring or averaging of density. The exceptional performance of the 3DLDM here highlights the benefit of its stable, noise-reducing objective, which allows it to preserve fine-grained soft-tissue contrast necessary for detailed abdominal anatomy.

**Figure 6:**
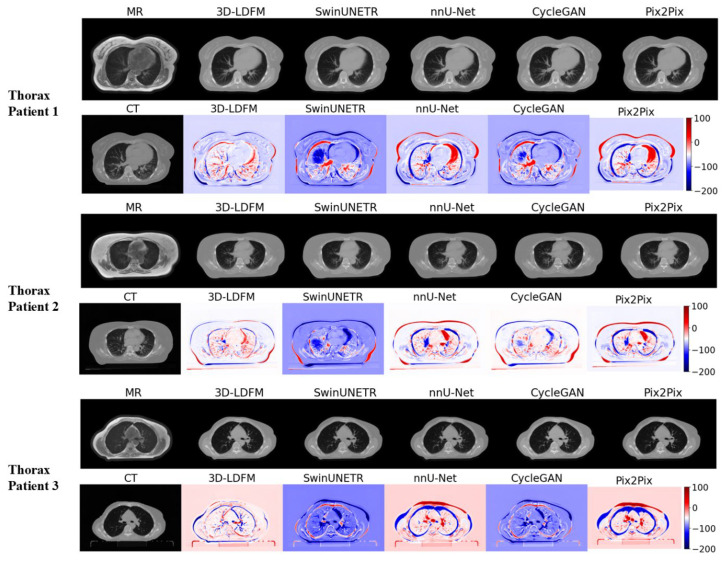
Thorax Slices within the Thoracic Volumes. Qualitative comparison and error maps for sCT generation on superior slices of the lower abdomen volumes that include lower thoracic structures.

The Thorax region presents unique challenges due to the large presence of air (lungs) and complex motion artifacts. Figure 6 demonstrates that the 3DLDM maintains superior structural preservation even in these challenging areas. Our model accurately delineates the low-density lung parenchyma and the bony rib cage without introducing the severe artifacts observed in the baselines. Examination of the error maps for the thorax reveals that the 3DLDM errors are highly localized and low in magnitude. Conversely, SwinUNeTr and Pix2Pix show substantial, diffuse blue and red regions across the lung and chest wall. The widespread blue error (overestimation of density) by baselines in the lung fields is a serious clinical concern, as it directly impacts dose calculation in radiotherapy planning. The consistent low error and sharp structural definition provided by the 3DLDM validate the efficiency of performing the diffusion process in the compressed latent space, which preserves volumetric consistency and prevents the introduction of non-physiologic anomalies seen in other architectures.

**Figure 7:**
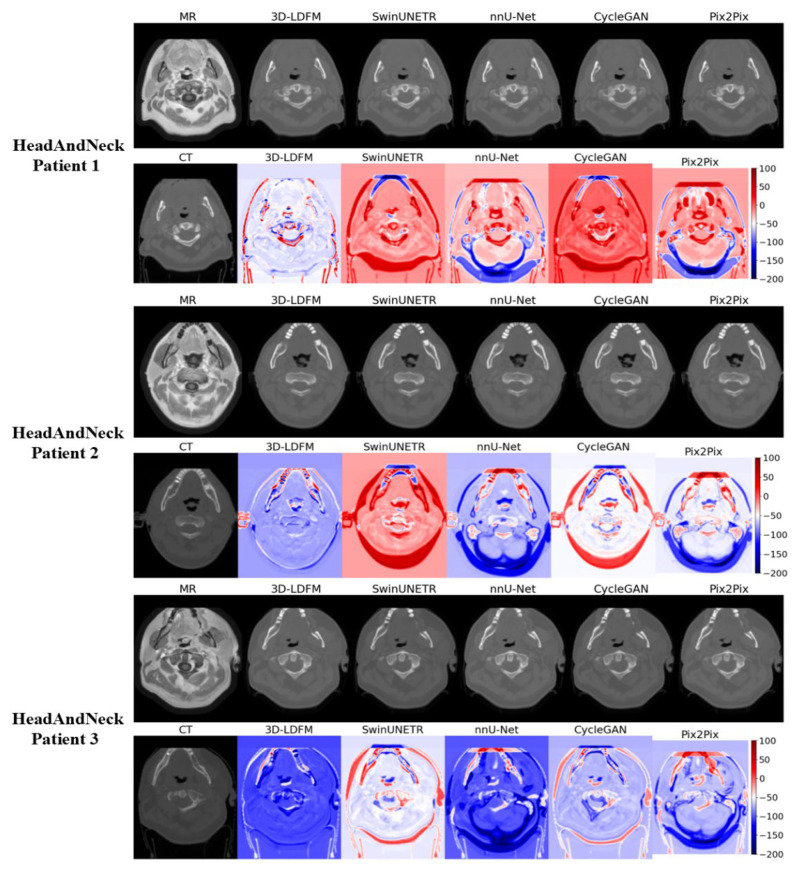
Head-and-Neck Slices within the Brain Volume. Qualitative comparison and error maps for sCT generation on inferior slices of the brain MRI/CT volumes that include head-and-neck anatomy.

As illustrated in Figure 7, the synthesis results for the Head and Neck (HN) region reveal pronounced differences in modeling complex bone and air structures. The images synthesized by our 3DLDM demonstrate the closest resemblance to the ground-truth CTs, particularly in accurately defining the high-density cortical bone structures (e.g., mandible and cervical spine) and the sharp boundaries of air cavities (e.g., pharynx and sinuses). This fidelity is emphatically confirmed by error maps (calculated as CT-sCT). The error maps for 3DLDM show minimal absolute deviation, with the error concentrated near the ±50 Hounsfield Unit (HU) range, indicating excellent HU accuracy. In sharp contrast, the error maps for the baseline models, particularly SwinUNeTr and CycleGAN, exhibit extensive areas of saturated red (>100HU) and deep blue (<−150HU). The high positive error (red) in these models suggests a significant underestimation of bone density (i.e., CT-sCT >0), while the high negative error (blue) indicates an overestimation of density in air-filled or soft-tissue regions. This failure by the baselines to accurately resolve sharp tissue interfaces highlights a common weakness of deterministic and adversarial models when dealing with high-contrast boundaries, a limitation successfully mitigated by the probabilistic, noise-conditioned approach of the 3DLDM.

Additionally, the consistent, low-magnitude, and localized error profile of the 3DLDM demonstrates that the stable, noise-reducing objective of the latent diffusion process successfully mitigates the structural inconsistencies and artifact generation that plague both deterministic (NNUNet) and adversarial (CycleGAN, Pix2Pix) models. Finally, the qualitative analysis (Figure 5, Figure 6 and Figure 7) strongly supports the conclusion that the 3DLDM is the most robust and accurate method for synthesizing clinically relevant CT volumes across multiple anatomical sites.

### 4.3. Proposed Model Benchmarking with State-of-the-Art Methods

To validate the effectiveness of our proposed 3D 3DLDM, we performed a comprehensive quantitative and qualitative comparison against several state-of-the-art methods in medical image synthesis. The selected baselines—SwinUNeTr (a transformer-based model), NNUNet (a high-performing U-Net variant), and CycleGAN (a leading unpaired GAN-based model)—represent the current breadth of approaches in the field. All models were trained and evaluated on identical splits of the SynthRAD2023 dataset to ensure a fair comparison. We report the mean and standard deviation of three widely accepted metrics (MAE, PSNR, SSIM) across three distinct anatomical regions: Abdomen, Head & Neck, and Thorax, as well as an average across all regions.

Table 2 summarizes the MAE results in Hounsfield Units (HU), where lower values indicate better agreement with the ground-truth CT. Across all anatomical regions, 3D-LDM achieved the lowest MAE, demonstrating superior voxel-wise intensity accuracy. In the Abdomen, 3D-LDM (57.24 HU) significantly outperformed SwinUNeTr (79.70 HU, *p* < 0.0001), nnU-Net (69.13 HU, *p* < 0.0001), and Pix2Pix (71.24 HU, *p* < 0.01), while the difference compared with CycleGAN (62.54 HU) was not statistically significant (*p* > 0.05). A similar pattern was observed in the Head & Neck region, where 3D-LDM (52.51 HU) achieved significantly lower error than SwinUNeTr (75.29 HU, *p* < 0.0001), nnU-Net (66.91 HU, *p* < 0.01), and Pix2Pix (69.33 HU, *p* < 0.01). In the Thorax, 3D-LDM (60.34 HU) again showed statistically significant improvements over SwinUNeTr (77.19 HU, *p* < 0.0001), nnU-Net (65.71 HU, *p* < 0.01), and Pix2Pix (72.39 HU, *p* < 0.01), with no significant difference relative to CycleGAN (57.91 HU). When averaged across all anatomical sites, 3D-LDM achieved the lowest overall MAE (56.44 HU), confirming its ability to produce more accurate synthetic CT attenuation values while reducing voxel-wise residual error.

As shown in Table 3, the proposed 3D-LDM achieved the highest PSNR across all anatomical regions (29.99 dB for Abdomen, 29.63 dB for Head & Neck, and 29.62 dB for Thorax), reflecting superior signal fidelity and reduced noise in the synthesized CT volumes. These improvements were statistically significant relative to all baseline models. In the Abdomen region, 3D-LDM outperformed SwinUNeTr (27.80 dB, *p* < 0.0001), nnU-Net (28.10 dB, *p* < 0.0001), CycleGAN (28.86 dB, *p* < 0.01), and Pix2Pix (27.92 dB, *p* < 0.0001). A similar pattern was observed for Head & Neck, where 3D-LDM achieved significantly higher PSNR than SwinUNeTr (*p* < 0.0001), nnU-Net (*p* < 0.01), CycleGAN (*p* < 0.05), and Pix2Pix (*p* < 0.0001). For Thorax, the performance gap remained statistically significant when compared with SwinUNeTr (*p* < 0.01), nnU-Net (*p* < 0.05), CycleGAN (*p* < 0.01), and Pix2Pix (*p* < 0.0001).

When averaged across all regions, 3D-LDM reached a PSNR of 29.73 ± 1.60 dB—substantially higher than SwinUNeTr (27.78 ± 2.06 dB, *p* < 0.0001), nnU-Net (28.46 ± 1.73 dB, *p* < 0.0001), CycleGAN (28.72 ± 1.61 dB, *p* < 0.01), and Pix2Pix (27.56 ± 1.95 dB, *p* < 0.0001). These results confirm that the observed improvements are not only numerically superior but statistically robust, demonstrating the consistent advantage of the diffusion-based framework for maintaining image quality across diverse anatomical regions.

As shown in Table 4, the proposed 3D-LDM achieved the highest SSIM values across all anatomical regions, indicating improved perceptual and structural fidelity in the synthesized CT volumes. In the Abdomen region, 3D-LDM reached an SSIM of 0.890, outperforming SwinUNeTr (0.839, *p* < 0.01), nnU-Net (0.873, *p* < 0.01), CycleGAN (0.886, n.s.), and Pix2Pix (0.847, *p* < 0.0001). For Head & Neck, 3D-LDM achieved 0.880, significantly higher than SwinUNeTr (0.833, *p* < 0.0001), nnU-Net (0.866, *p* < 0.05), CycleGAN (0.875, n.s.), and Pix2Pix (0.845, *p* < 0.0001). A similar trend was observed in the Thorax region, where 3D-LDM (0.885) exceeded SwinUNeTr (0.849, *p* < 0.01), nnU-Net (0.869, *p* < 0.05), CycleGAN (0.869, *p* < 0.05), and Pix2Pix (0.844, *p* < 0.0001).

When averaged across all regions, 3D-LDM achieved the highest overall SSIM (0.885), with statistically significant improvements over SwinUNeTr (0.840, *p* < 0.0001), nnU-Net (0.869, *p* < 0.01), CycleGAN (0.876, *p* < 0.05), and Pix2Pix (0.845, *p* < 0.0001). These findings demonstrate that the diffusion-based approach preserves structural similarity more effectively than CNN, GAN, or transformer-based baselines, highlighting its suitability for clinical applications where anatomical fidelity is essential.

### 4.4. Computational Efficiency

To evaluate computational scalability, we measured inference time per 3D volume across anatomical regions. The proposed 3D-LDM required 28.43 s per volume depending on region, with the thorax being the most computationally demanding due to its large air–bone transitions. On average, 3D-LDM was faster than GAN-based pipelines (CycleGAN: 23.83 s, Pix2Pix: 18.22 s) and substantially more efficient than voxel-space diffusion models reported in prior studies, while delivering significantly improved fidelity (Table 2, Table 3 and Table 4). Although nnU-Net achieves lower inference time, it does so at the cost of higher MAE and lower PSNR and SSIM, demonstrating the classical trade-off between inference speed and structural accuracy.

## 5. Discussion

This study’s successful implementation of a 3D 3DLDM for cross-modal medical image synthesis carries significant implications for both generative modeling theory and clinical practice. The principal theoretical contribution is the validation of LDMs as a superior and scalable model for high-dimensional volumetric synthesis, effectively resolving the limitations of prior methods [25,26,29,30]. The 3DLDM overcomes the chronic instability and mode collapse endemic to GANs, allowing it to accurately learn and represent the full distribution of complex Hounsfield Unit (HU) values, particularly those associated with high-contrast tissues like bone and air. This improved generative stability directly yields a more reliable and anatomically consistent output. Furthermore, the two-stage architecture compressing the 3D volume into a low-dimensional latent space, establishes a crucial principle for scalable 3D synthesis, mitigating the prohibitive memory and runtime costs associated with performing diffusion directly in the voxel space [35].

The proposed framework has important implications for clinical workflows, particularly in radiation oncology and multi-modal imaging. By achieving high volumetric fidelity and low attenuation error—supported by low MAE and spatially localized error maps—our 3D-LDM enables the possibility of MR-only radiotherapy planning, reducing or potentially eliminating the need for an additional planning CT scan and thereby avoiding unnecessary ionizing radiation exposure. Synthetic CT technology has already demonstrated clinical feasibility for treatment planning in several anatomical regions, and our work further advances this direction by providing a unified, multi-region diffusion-based solution. End-to-end clinical evaluation has also shown that AI-based sCT models can achieve acceptable gamma passing rates in brain and pelvis treatments [36]. The generated sCTs are sufficiently reliable for dose calculation and organ-at-risk delineation because of their precise preservation of structural integrity (especially in bony and soft-tissue boundaries) [37]. Our results further advance the field by providing a unified, diffusion-based model validated across head & neck, thorax, and abdomen regions. The 3D-LDM also offers strong utility for data augmentation, enabling the synthesis of anatomically diverse CT volumes that can improve downstream diagnostic and anomaly detection models, particularly for rare pathologies or small datasets.

Despite its overall strong performance, the model exhibited higher errors in a small subset of cases. Most failures were associated with metal implants, motion artifacts, or unusual anatomy (e.g., surgical alterations), which introduced MRI distortions and resulted in localized HU inaccuracies—especially near bone–air boundaries. In a few outlier cases, blurred cortical interfaces or localized intensity shifts were observed, likely due to limited representation of such patterns in the training set. These cases highlight well-known failure modes in synthetic CT generation and indicate that image quality and anatomical variability remain important factors. Future work will explore artifact-aware training, uncertainty estimation, and integration of dose-aware objectives to improve robustness in these edge cases.

## 6. Conclusions

In this study, we successfully introduced and validated a 3D latent diffusion model for high-fidelity MRI-to-CT synthesis, directly addressing the critical clinical need for non-radiating, accurate cross-modal imaging. Our two-stage framework, which leverages latent space compression to maintain computational efficiency while applying the robust, noise-conditioned objective of diffusion models, demonstrated significant advantages over state-of-the-art GAN- and CNN-based methods. Quantitatively, the 3DLDM achieved superior performance across all anatomical sites (Head & Neck, Thorax, Abdomen) in terms of accuracy (lowest MAE) and structural preservation (highest PSNR and SSIM). Qualitatively, the model mitigated the common failure modes of baselines, producing synthetic CT volumes with minimal, localized error, particularly around complex, high-contrast interfaces like bone and air. This work establishes the 3DLDM as a scalable, stable, and highly accurate solution for volumetric medical image synthesis. Lastly, our approach provides a critical technological foundation for realizing MR-only radiotherapy planning and accelerating the adoption of multi-modal, radiation-free imaging solutions in the clinical environment. Future work will extend this study toward direct clinical validation by incorporating dose-aware optimization, performing full dosimetric evaluation, and computing Dose–Volume Histograms (DVHs) using clinical treatment planning systems to quantify the impact of synthetic CT on radiotherapy dose calculation.

## Figures and Tables

**Table 1:** Dataset distribution for Task 1, summarizing the number of paired MRI–CT volumes in the training and test sets across head and neck, thorax, and abdominal cohorts.

Task 1	Head and Neck	Thorax	Abdominal	All
Train	177	146	140	463
Test	44	36	35	115
All	221	182	175	578

**Table 2:** Mean Absolute Error of Synthetic CT Generation. All units in (HU, ↓).

Region	3D-LDM (Ours)	SwinUNeTr	NNUNet	CycleGAN	Pix2Pix
Abdomen (AB)	57.24 (±18.48)	79.70 (±12.07) ***	69.13 (±28.87)	62.54 (±20.19)	71.24 (±16.44) **
Head & Neck (HN)	52.51 (±21.56)	75.29 (±11.44) ***	66.91 (±23.42) **	59.92 (±17.08)	69.33 (±15.31) **
Thorax (TH)	60.34 (±24.11)	77.19 (±11.34) ***	65.71 (±25.49)	57.91 (±15.18)	72.39 (±15.98) **
All	56.44 (±21.63)	77.23 (±11.65) ***	67.20 (±25.63) **	60.07 (±17.47)	70.89 (±15.78) ***

Statistical significance was assessed using paired two-sided *t*-tests versus 3D-LDFM. Significance levels: *** *p* < 0.0001, ** *p* < 0.01, and * *p* < 0.05.

**Table 3:** Quantitative Comparison of Image Quality by Peak Signal-to-Noise Ratio (dB).

Region	3D-LDM (Ours)	SwinUNeTr	NNUNet	CycleGAN	Pix2Pix
AB	29.99 (±1.58)	27.80 (±1.77) ***	28.10 (±1.70) ***	28.86 (±1.55) **	27.92 (±2.22) ***
HN	29.63 (±1.68)	27.63 (±2.42) ***	28.52 (±1.65) **	28.81 (±1.76) *	27.46 (±1.72) ***
TH	29.62 (±1.54)	27.94 (±1.89) **	28.72 (±1.83) *	28.47 (±1.47) **	27.35 (±1.94) ***
All	29.73 (±1.60)	27.78 (±2.06) ***	28.46 (±1.73) ***	28.72 (±1.61) ***	27.56 (±1.95) ***

Statistical significance was assessed using paired two-sided *t*-tests versus 3D-LDFM. Significance levels: *** *p* < 0.0001, ** *p* < 0.01, and * *p* < 0.05.

**Table 4:** Quantitative Comparison of Structural Similarity by SSIM (↑).

Region	3D-LDM (Ours)	SwinUNeTr	NNUNet	CycleGAN	Pix2Pix
AB	0.890 (±0.0327)	0.839 (±0.0587) **	0.873 (±0.0363)	0.886 (±0.0280)	0.847 (±0.037) ***
HN	0.880 (±0.0333)	0.833 (±0.0536) ***	0.866 (±0.0317) *	0.875 (±0.0336)	0.845 (±0.038) ***
TH	0.885 (±0.0264)	0.849 (±0.047) **	0.869 (±0.0338) *	0.869 (±0.0349) *	0.844 (±0.040) ***
All	0.885 (±0.0310)	0.840 (±0.0532) ***	0.869 (±0.0336) **	0.876 (±0.0328) *	0.845 (±0.038) ***

Statistical significance was assessed using paired two-sided *t*-tests versus 3D-LDFM. Significance levels: *** *p* < 0.0001, ** *p* < 0.01, and * *p* < 0.05.

## Data Availability

The data supporting the findings of this study are publicly available on Zenodo (https://doi.org/10.5281/zenodo.7260705) under the SynthRAD2023 collection (Accessed: 19 January 2025). No additional data were generated or analyzed beyond those reported in this study.

## Abbreviations

The following abbreviations are used in this manuscript:

3D	Three-Dimensional
AB	Abdomen (Anatomical Region)
AMP	Automatic Mixed Precision
CNN	Convolutional Neural Network
CT	Computed Tomography
DDPM	Denoising Diffusion Probabilistic Model
DSC	Dice Similarity Coefficient
GAN	Generative Adversarial Network
HN	Head and Neck (Anatomical Region)
HU	Hounsfield Unit
IQR	Interquartile Range
LDM	Latent Diffusion Model
MAE	Mean Absolute Error
MRI	Magnetic Resonance Imaging
MSE	Mean Squared Error
PSNR	Peak Signal-to-Noise Ratio
sCT	Synthetic Computed Tomography
SSIM	Structural Similarity Index Measure
TH	Thorax (Anatomical Region)
VAE	Variational Autoencoder

## References

[1] Kitson S.L. (2024). Modern Medical Imaging and Radiation Therapy.

[2] Lazaros K., Adam S., Krokidis M.G., Exarchos T., Vlamos P., Vrahatis A.G. (2025). Non-invasive biomarkers in the era of big data and machine learning. Sensors.

[3] Bahloul M.A., Jabeen S., Benoumhani S., Alsaleh H.A., Belkhatir Z., Al-Wabil A. (2024). Advancements in synthetic CT generation from MRI: A review of techniques, and trends in radiation therapy planning. J. Appl. Clin. Med. Phys..

[4] Lother D., Robert M., Elwood E., Smith S., Tunariu N., Johnston S.R., Parton M., Bhaludin B., Millard T., Downey K. (2023). Imaging in metastatic breast cancer, CT, PET/CT, MRI, WB-DWI, CCA: Review and new perspectives. Cancer Imaging.

[5] Mahdi M.A., Ahamad S., Saad S.A., Dafhalla A., Alqushaibi A., Qureshi R. (2024). Enhancing Predictive Accuracy for Recurrence-Free Survival in Head and Neck Tumor: A Comparative Study of Weighted Fusion Radiomic Analysis. Diagnostics.

[6] Goyal M.K., Chaturvedi R. (2023). Synthetic data revolutionizes rare disease research: How large language models and generative AI are overcoming data scarcity and privacy challenges. Int. J. Recent Innov. Trends Comput. Commun..

[7] Zhu E., Muneer A., Zhang J., Xia Y., Li X., Zhou C., Heymach J.V., Wu J., Le X. (2025). Progress and challenges of artificial intelligence in lung cancer clinical translation. npj Precis. Oncol..

[8] Banerjee J., Taroni J.N., Allaway R.J., Prasad D.V., Guinney J., Greene C. (2023). Machine learning in rare disease. Nat. Methods.

[9] Decherchi S., Pedrini E., Mordenti M., Cavalli A., Sangiorgi L. (2021). Opportunities and challenges for machine learning in rare diseases. Front. Med..

[10] Shin H.-C., Tenenholtz N.A., Rogers J.K., Schwarz C.G., Senjem M.L., Gunter J.L., Andriole K.P., Michalski M. Medical image synthesis for data augmentation and anonymization using generative adversarial networks. Proceedings of the International Workshop on Simulation and Synthesis in Medical Imaging.

[11] Frid-Adar M., Diamant I., Klang E., Amitai M., Goldberger J., Greenspan H. (2018). GAN-based synthetic medical image augmentation for increased CNN performance in liver lesion classification. Neurocomputing.

[12] Ibrahim M., Al Khalil Y., Amirrajab S., Sun C., Breeuwer M., Pluim J., Elen B., Ertaylan G., Dumontier M. (2025). Generative AI for synthetic data across multiple medical modalities: A systematic review of recent developments and challenges. Comput. Biol. Med..

[13] Yang Q., Li N., Zhao Z., Fan X., Chang E.I.-C., Xu Y. (2020). MRI cross-modality image-to-image translation. Sci. Rep..

[14] Lei Y., Harms J., Wang T., Liu Y., Shu H.K., Jani A.B., Curran W.J., Mao H., Liu T., Yang X. (2019). MRI-only based synthetic CT generation using dense cycle consistent generative adversarial networks. Med. Phys..

[15] Armanious K., Jiang C., Fischer M., Küstner T., Hepp T., Nikolaou K., Gatidis S., Yang B. (2020). MedGAN: Medical image translation using GANs. Comput. Med. Imaging Graph..

[16] Roberts M., Hinton G., Wells A.J., Van Der Veken J., Bajger M., Lee G., Liu Y., Chong C., Poonnoose S., Agzarian M. (2023). Imaging evaluation of a proposed 3D generative model for MRI to CT translation in the lumbar spine. Spine J..

[17] Bahrami A., Karimian A., Fatemizadeh E., Arabi H., Zaidi H. (2020). A new deep convolutional neural network design with efficient learning capability: Application to CT image synthesis from MRI. Med. Phys..

[18] Kaiser B., Albarqouni S. (2019). MRI to CT translation with GANs. arXiv.

[19] Emami H., Dong M., Nejad-Davarani S.P., Glide-Hurst C.K. SA-GAN: Structure-aware GAN for organ-preserving synthetic CT generation. Proceedings of the International Conference on Medical Image Computing and Computer-Assisted Intervention.

[20] van der Ouderaa T.F., Worrall D.E., van Ginneken B. (2019). Chest CT super-resolution and domain-adaptation using memory-efficient 3D reversible GANs. arXiv.

[21] Chartsias A., Joyce T., Dharmakumar R., Tsaftaris S.A. Adversarial image synthesis for unpaired multi-modal cardiac data. Proceedings of the International Workshop on Simulation and Synthesis in Medical Imaging.

[22] Sherwani M.K., Gopalakrishnan S. (2024). A systematic literature review: Deep learning techniques for synthetic medical image generation and their applications in radiotherapy. Front. Radiol..

[23] Muneer A., Waqas M., Saad M.B., Showkatian E., Bandyopadhyay R., Xu H., Li W., Chang J.Y., Liao Z., Haymaker C. (2025). From Classical Machine Learning to Emerging Foundation Models: Review on Multimodal Data Integration for Cancer Research. arXiv.

[24] Ronneberger O., Fischer P., Brox T. U-net: Convolutional networks for biomedical image segmentation. Proceedings of the International Conference on Medical Image Computing and Computer-Assisted Intervention.

[25] Jafarpour F. (2024). Synthetic CT Generation from MR Images: A U-Net Deep Learning Approach. https://thesis.unipd.it/handle/20.500.12608/73701.

[26] Liu Y., Chen A., Shi H., Huang S., Zheng W., Liu Z., Zhang Q., Yang X. (2021). CT synthesis from MRI using multi-cycle GAN for head-and-neck radiation therapy. Comput. Med. Imaging Graph..

[27] Zhu J.-Y., Park T., Isola P., Efros A.A. Unpaired image-to-image translation using cycle-consistent adversarial networks. Proceedings of the IEEE International Conference on Computer Vision.

[28] Islam S., Aziz M.T., Nabil H.R., Jim J.R., Mridha M.F., Kabir M.M., Asai N., Shin J. (2024). Generative adversarial networks (GANs) in medical imaging: Advancements, applications, and challenges. IEEE Access.

[29] Meng M. (2025). Modeling Fine-grained Long-range Visual Dependency for Deep Learning-based Medical Image Analysis. Ph.D. Thesis.

[30] Pan S., Abouei E., Wynne J., Chang C.W., Wang T., Qiu R.L., Li Y., Peng J., Roper J., Patel P. (2024). Synthetic CT generation from MRI using 3D transformer-based denoising diffusion model. Med. Phys..

[31] Pinaya W.H., Tudosiu P.-D., Dafflon J., Da Costa P.F., Fernandez V., Nachev P., Ourselin S., Cardoso M.J. Brain imaging generation with latent diffusion models. Proceedings of the MICCAI Workshop on Deep Generative Models.

[32] Kui X., Liu B., Sun Z., Li Q., Zhang M., Liang W., Zou B. (2025). Med-LVDM: Medical latent variational diffusion model for medical image translation. Biomed. Signal Process. Control..

[33] Rombach R., Blattmann A., Lorenz D., Esser P., Ommer B. High-resolution image synthesis with latent diffusion models. Proceedings of the IEEE/CVF Conference on Computer Vision and Pattern Recognition.

[34] Huijben E.M., Terpstra M.L., Pai S., Thummerer A., Koopmans P., Afonso M., Van Eijnatten M., Gurney-Champion O., Chen Z., Zhang Y. (2024). Generating synthetic computed tomography for radiotherapy: SynthRAD2023 challenge report. Med. Image Anal..

[35] Kim J., Park H. Adaptive latent diffusion model for 3d medical image to image translation: Multi-modal magnetic resonance imaging study. Proceedings of the IEEE/CVF Winter Conference on Applications of Computer Vision.

[36] Parchur A., Paulson E., Ahunbay E. (2025). End-to-End Clinical Evaluation Testing of Synthetic CT for MRI-Only Brain and Pelvis Radiotherapy. Int. J. Radiat. Oncol. Biol. Phys..

[37] Fusella M., Andres E.A., Villegas F., Milan L., Janssen T., Dal Bello R., Garibaldi C., Placidi L., Cusumano D. (2024). Results of 2023 survey on the use of synthetic computed tomography for magnetic resonance Imaging-only radiotherapy: Current status and future steps. Phys. Imaging Radiat. Oncol..

